# Epidemiology and Outcomes of Acute Pulmonary Embolism in Seychelles: A National Cohort Study With a Subgroup Analysis of Systemic Thrombolysis Efficacy and Safety in Intermediate-Risk Pulmonary Embolism

**DOI:** 10.7759/cureus.113190

**Published:** 2026-07-22

**Authors:** Mahmoud F Hassanein, Alaa M Ebrahim, Ahmed L Ibrahim, Ramprasath Anbazhagan, Maarej Rizvi, Nivetha Pandiyan, Mahmoud S Ahmed, Stephanie A Joseph, Clara W Omath, Chukwuemeka S Anadozie, Laura V Gayon, Marie C De Lacoudraye, Lucile Decomarmond

**Affiliations:** 1 Internal Medicine, Seychelles Hospital, Victoria, SYC; 2 Cardiology, Seychelles Hospital, Victoria, SYC; 3 Radiology, Seychelles Hospital, Victoria, SYC; 4 Critical Care Medicine, Seychelles Hospital, Victoria, SYC; 5 Pharmacology and Therapeutics, Seychelles Hospital, Victoria, SYC

**Keywords:** anticoagulation, incidence in seychelles, intermediate-risk pulmonary embolism, pulmonary embolism, respiratory distress, right-sided heart strain, submassive pulmonary embolism, systemic thrombolysis, thrombolysis window time, thrombolytic agent

## Abstract

Background

Pulmonary embolism (PE) is an important cause of cardiovascular morbidity and mortality worldwide. Data from Small Island Developing States (SIDS) in Africa are limited. This study describes the epidemiology of acute PE in Seychelles and examines outcomes associated with different management approaches in patients with intermediate-risk PE.

Methods

This retrospective observational cohort study was conducted at Seychelles Hospital, Victoria, the national tertiary referral hospital and principal public healthcare facility in the Republic of Seychelles. We conducted a retrospective observational cohort study of all adults with confirmed acute PE managed in Seychelles between January 1, 2019, and December 31, 2025. Temporal trends in incidence, mortality, and hospital length of stay were evaluated. An exploratory subgroup analysis was performed in patients with intermediate-risk PE managed between January 1, 2019, and May 31, 2026, to assess clinical outcomes across this heterogeneous cohort. Patients were grouped according to treatment strategy: systemic thrombolysis followed by anticoagulation (Group A; n = 48) or anticoagulation alone (Group B; n = 12). Clinical, physiological, echocardiographic, and safety outcomes were assessed.

Results

A total of 389 patients with acute PE were identified during the study period. Females accounted for 231 (59.4%) cases and males for 158 (40.6%). Annual case numbers increased from 36 in 2019 to 84 in 2024 before decreasing to 69 in 2025. Overall crude mortality was 7.5% (29/389), ranging from 20.0% in 2020 to 0.0% in 2025. Among patients with intermediate-risk PE, the observed survival was higher in the thrombolysis group than in the anticoagulation-only group (47/48 (97.9%) vs. 8/12 (66.7%); p = 0.0045). In the thrombolysis group, 32 patients out of the 48 received tenecteplase in myocardial infarction dose as a thrombolytic agent and 16 patients received alteplase. Significant improvements were observed following thrombolysis in oxygen saturation (p < 0.001), PaO_2_/FiO_2_ ratio (p < 0.00001), and tricuspid annular plane systolic excursion (TAPSE; p = 0.0104). Right ventricular dilatation decreased on follow-up echocardiography (p = 0.0156), and McConnell’s sign resolved in all patients with available follow-up studies. One patient (2.1%) experienced intracranial hemorrhage after thrombolysis. Minor bleeding events included self-limited haematuria (n = 11) and gum bleeding (n = 3).

Conclusion

PE represents a substantial clinical burden in Seychelles. In this retrospective cohort, systemic thrombolysis in selected patients with intermediate-risk PE demonstrated a favorable association with physiological and echocardiographic outcomes, as well as lower observed mortality, compared to anticoagulation alone. These exploratory findings highlight important clinical trends, although the inherent retrospective design limits direct causal evaluations of treatment efficacy. Tenecteplase in the myocardial infarction dosage was associated with good outcomes in treating intermediate-risk PE. We observed successful thrombolysis outcomes within a window time up to seven days.

## Introduction

Pulmonary embolism (PE) is one of the leading causes of cardiovascular death worldwide and remains an important cause of hospital admission and mortality [1-3]. Clinical presentation ranges from incidental findings on imaging to circulatory collapse and sudden death. Risk stratification is central to management and is commonly based on haemodynamic status, right ventricular (RV) function, and markers of myocardial injury. Patients are generally classified as low-risk, intermediate-risk, or high-risk (previously non-massive, submassive, and massive, respectively) [4-6].

Low-risk PE is usually managed with anticoagulation alone. High-risk PE is characterized by haemodynamic instability and requires urgent reperfusion therapy. Intermediate-risk PE includes patients who remain haemodynamically stable but have evidence of RV dysfunction, myocardial injury, or both. Management of this group remains debated. Although systemic thrombolysis may improve RV function and reduce haemodynamic deterioration, it is associated with an increased risk of major bleeding, including intracranial haemorrhage [7-10].

Data describing PE epidemiology in African countries are limited, and information from Small Island Developing States is particularly scarce. Seychelles is an island nation with a centralised public healthcare system. Advanced reperfusion techniques, such as catheter-directed thrombectomy and surgical pulmonary embolectomy, are not available locally, making systemic pharmacological treatment the main reperfusion strategy for patients requiring escalation of care.

Between 2019 and 2025, two events affected healthcare delivery and data collection in Seychelles. The COVID-19 pandemic influenced hospital activity and thromboembolic disease patterns worldwide. In addition, the national transition from paper records to an electronic medical record (EMR) system in late 2024 changed the way clinical information was recorded and retrieved.

Tenecteplase was added to the Seychelles National Essential Medication List in mid-2024 and was subsequently used in selected patients with intermediate-risk PE according to local hospital practice. Because it can be administered as a single intravenous bolus, it offered an alternative to alteplase in routine clinical care.

The primary objective of this retrospective study was to assess the observed associations between treatment strategy, specifically systemic thrombolysis versus anticoagulation alone, and short-term clinical outcomes in patients diagnosed with intermediate-risk PE in Seychelles. Secondarily, we sought to conduct an exploratory comparison within this intermediate-risk cohort. This exploratory analysis aims to observe outcome trends while qualitatively considering variables such as age, underlying etiology, contraindications, treatment era, and the specific thrombolytic agent utilized.

## Materials and methods

Study objectives

Primary Epidemiological Objective

To describe the nationwide epidemiology, annual incidence, hospital average length of stay, and mortality rate of acute PE in Seychelles over a seven-year period (January 2019 to December 2025), evaluating how broader healthcare challenges (such as the COVID-19 pandemic) and system-wide improvements (such as the 2024 implementation of EMRs) influenced diagnostic trends, patient tracking, and overall clinical characteristics.

Primary Clinical Objective

This study aimed to evaluate the observed associations between the chosen therapeutic strategy (systemic thrombolysis plus anticoagulation (Group A) vs. anticoagulation alone (Group B)) and key clinical, physiological, and safety outcomes within a retrospective cohort of intermediate-risk PE patients in Seychelles [1,2].

Exploratory Subgroup Objective

This study also aimed to conduct an exploratory comparison within the intermediate-risk cohort. Given the clinical heterogeneity of intermediate-risk PE, this analysis does not attempt a fully adjusted causal evaluation of efficacy; rather, it serves as an exploratory assessment to observe outcomes while accounting for variations in patient age, underlying etiology, provoking factors, clinical comorbidities, bleeding risks, treatment era, and the thrombolytic agent administered.

Study design and setting

**Figure 1:**
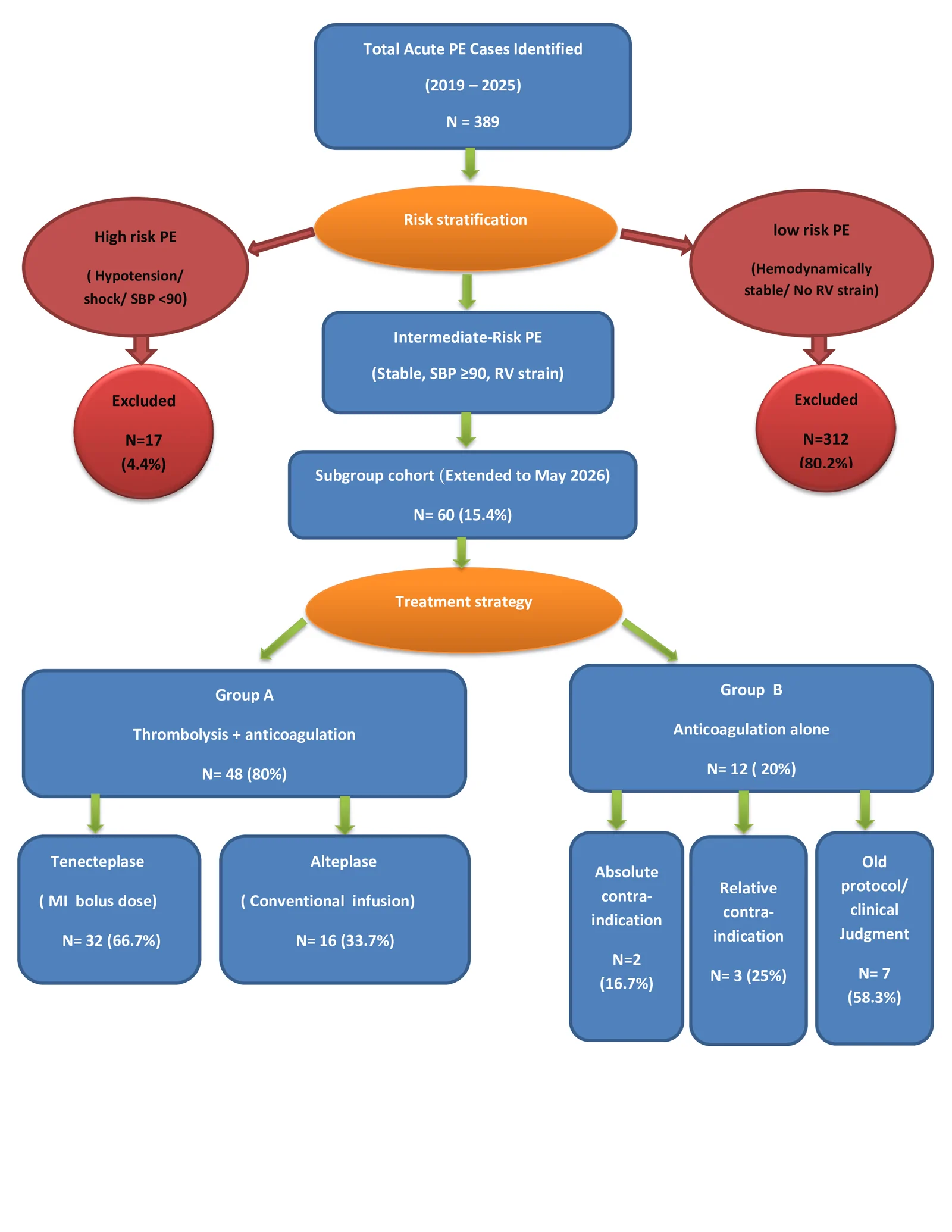
Flowchart of patient screening, risk stratification, and group allocation

This retrospective observational cohort study was conducted at Seychelles Hospital, Victoria, the national tertiary referral hospital and principal public healthcare facility in the Republic of Seychelles. The study included all adult patients diagnosed with acute PE between January 1, 2019, and December 31, 2025, for the epidemiological analysis. For the intermediate-risk PE subgroup analysis, data collection was extended to May 31, 2026. Clinical data were obtained from hospital admission records, patient medical records, imaging reports, intensive care unit (ICU) databases, and the hospital statistics department (Figure 1).

Study population and risk stratification

Adult patients aged 18 years or older with objectively confirmed acute PE were eligible for inclusion. Diagnosis was established primarily by computed tomography pulmonary angiography (CTPA). Patients were stratified according to haemodynamic status and evidence of right ventricular (RV) dysfunction, based on contemporary guideline recommendations [1,2].

High-Risk PE

It is defined as persistent hypotension (systolic blood pressure <90 mmHg), cardiogenic shock, or the need for vasopressor support.

Low-Risk PE

It is defined as haemodynamic stability without evidence of RV dysfunction or other markers of clinical severity.

Intermediate-Risk PE

It is defined as haemodynamic stability (systolic blood pressure ≥90 mmHg without vasopressor support) with objective evidence of RV dysfunction on echocardiography, CT imaging, or electrocardiography [1,2].

Inclusion and exclusion criteria

Patients were included in the subgroup analysis if they met criteria for intermediate-risk PE and were aged 18 years or older. Patients classified as high-risk or low-risk PE were excluded from the comparative treatment analysis.

Data quality considerations

Two healthcare system events affected data collection during the study period. First, the COVID-19 pandemic in 2020 resulted in incomplete documentation and reduced availability of some clinical records. Second, the national transition from paper-based records to an electronic medical record (EMR) system in late 2024 altered data capture processes. Another encountered challenge was the frequent shortage of certain biomarkers (e.g., troponin and pro BNP) due to difficult, timely supply to a small island country; for this reason, it was excluded from the study parameters. Where possible, records spanning these periods were manually verified using both paper and electronic sources.

Treatment strategies

Patients with intermediate-risk PE were managed according to institutional practice and clinician judgement.

Group A (systemic thrombolysis plus anticoagulation) included patients treated with systemic thrombolysis followed by long-term anticoagulation (n = 48). Alteplase was used during the earlier study period at a dose of 100 mg IV infused over two hours, whereas tenecteplase became increasingly used after its inclusion in the Seychelles National Essential Medication List in mid-2024. Tenecteplase was administered using weight-adjusted single-bolus dosing derived from established myocardial infarction protocols [11]: <60 kg: 30 mg; 60-69 kg: 35 mg; 70-79 kg: 40 mg; 80-89 kg: 45 mg; and ≥90 kg: 50 mg.

Long-term anticoagulation consisted primarily of rivaroxaban or warfarin.

Group B (Anticoagulation alone) included patients managed without thrombolysis (n = 12), who received a therapeutic dose of rivaroxaban 15 mg BD for 15 days, then 20 mg OD for three to 12 months, or enoxaparin 1 mg/kg/d BD for at least five days plus warfarin 5-10 mg OD for three to 12 months. Decisions to withhold thrombolysis were based on clinician assessment, contraindications to thrombolytic therapy, bleeding risk, or institutional practice patterns.

Monitoring and follow-up

Patients undergoing systemic thrombolysis were monitored in the ICU for 24-48 hours via continuous ECG, blood pressure monitoring, pulse oximetry, neurological checks, and serial laboratory tests.

Baseline assessments, including chest CT angiography, ECG, echocardiography, ABG (for PaO_2/FiO2 ratio), and vital signs, were typically completed within 24 hours prior to thrombolysis. For patients with delayed thrombolysis, vital signs were continuously monitored alongside serial ECGs and ABGs.

All clinical, laboratory, and imaging investigations were repeated 24-48 hours post-thrombolysis upon step-down to the general ward to evaluate treatment response. Vital signs monitoring continued until hospital discharge

Outcomes

The primary outcomes were all-cause in-hospital mortality, 30-day mortality, and clinical deterioration. Secondary outcomes included physiological response, echocardiographic changes, length of hospital stay, and bleeding complications. Bleeding events were classified as major or minor according to clinical severity [12].

Statistical analysis

Continuous variables are expressed as mean ± standard deviation (SD) or median (interquartile range (IQR)) based on distribution normality. Categorical items are detailed as absolute counts and paired percentages (N, %).

Pre- and post-thrombolysis physiological markers were analyzed using two-tailed paired t-tests (t-statistic reported). Proportional changes in paired categorical variables were evaluated via McNemar’s test (χ2 reported). Survival and mortality rates between cohorts were compared using Fisher’s exact test. Statistical significance was defined as p < 0.05, with higher thresholds noted where applicable (p < 0.001, p < 0.00001). All statistical evaluations were conducted using verified analytical software.

## Results

National epidemiology and trends (2019-2025)

Between January 1, 2019, and December 31, 2025, a total of 389 patients with acute PE were identified in Seychelles. Annual case numbers increased from 36 in 2019 to 84 in 2024 before decreasing to 69 in 2025 (Table 1). Among all patients, 231 (59.4%) were female and 158 (40.6%) were male.

**Table 1:** Annual breakdown of pulmonary embolism epidemiology in Seychelles (2019–2025). Data are presented as absolute counts (N), percentages (%), or arithmetic means ± SD. Statistical comparisons across years are descriptive. Significance threshold set at p < 0.05.

Year	Male cases, N (%)	Female cases, N (%)	Total cases, N	Absolute mortality, N	Annual mortality rate (%)	Average length of stay (days, mean ± SD)	Data integrity / system note
2019	12 (33.3%)	24 (66.7%)	36	4	11.1%	4.08 ±1.12	Baseline paper records
2020	21 (60.0%)	14 (40.0%)	35	7	20.0%	6.14 ±2.45	Incomplete data (COVID-19 wave)
2021	13 (35.1%)	24 (64.9%)	37	4	10.8%	9.33 ±3.11	Post-pandemic stabilization
2022	17 (34.7%)	32 (65.3%)	49	8	16.3%	7.58 ±2.04	Baseline paper records
2023	36 (45.6%)	43 (54.4%)	79	2	2.5%	8.84 ±2.89	Baseline paper records
2024	32 (38.1%)	52 (61.9%)	84	4	4.8%	5.33 ±1.67	EMR transition phase
2025	24 (34.8%)	45 (65.2%)	69	0	0.0%	3.84 ±0.95	Full electronic record deployment
Total	158 (40.6%)	231 (59.4%)	389	29	7.46% (Avg)	6.45 ± 2.15 (Avg)	—

**Figure 2:**
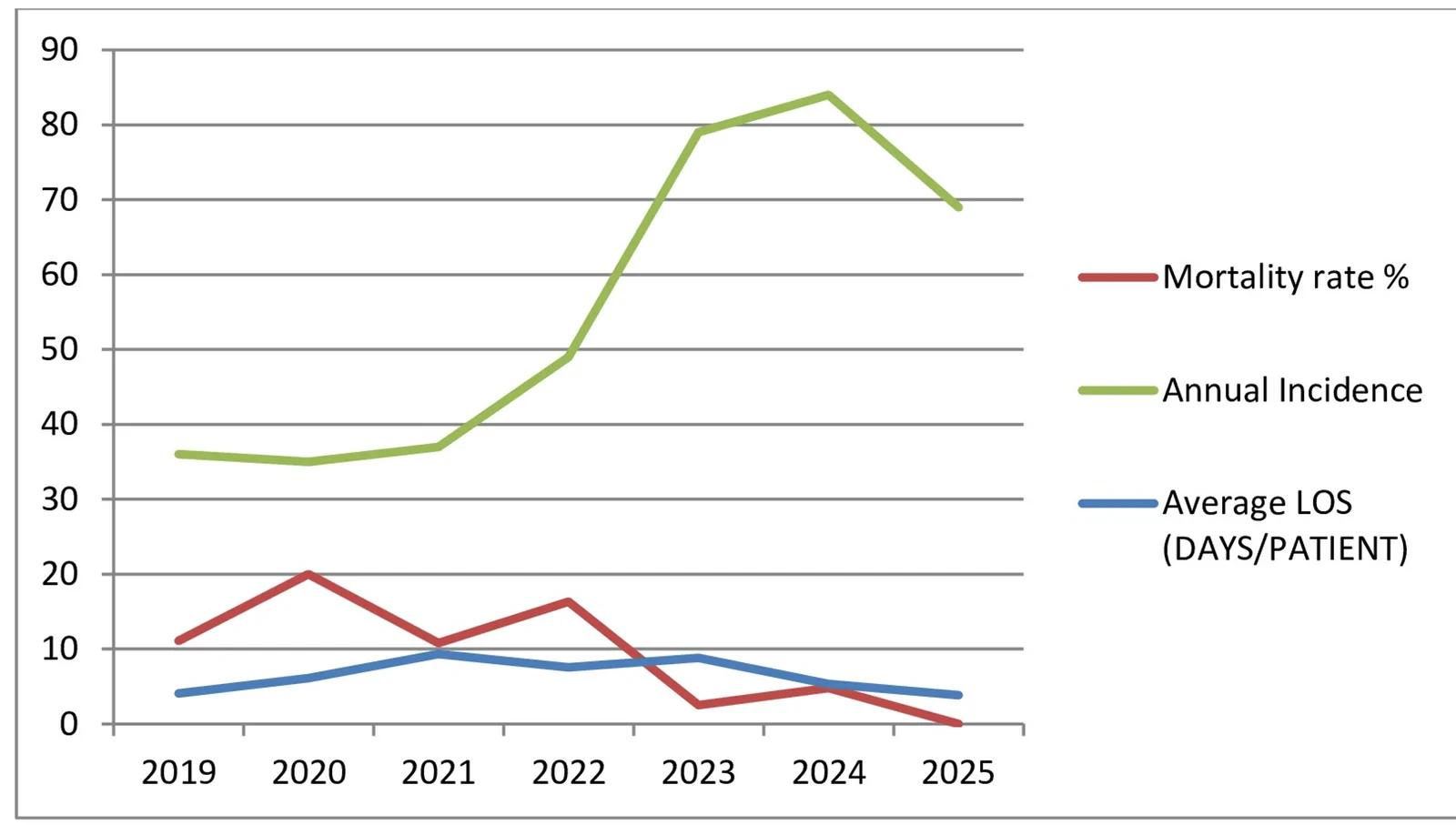
Correlation between annual incidence, mortality rate, and average length of stay of pulmonary embolism in Seychelles.

Overall mortality during the study period was 29 of 389 patients (7.5%). Annual mortality dropped from 20.0% in 2020 to 0.0% in 2025. Mean hospital length of stay dropped from 9.33 days in 2021 to 3.84 days in 2025 (Figure 2).

Hypertension was the most frequently documented comorbidity (n = 43), followed by diabetes mellitus (n = 16) and chronic heart failure (n = 8).

Submassive (intermediate-risk) PE subgroup analysis (N = 60 patients)

Baseline Characteristics

**Figure 3:**
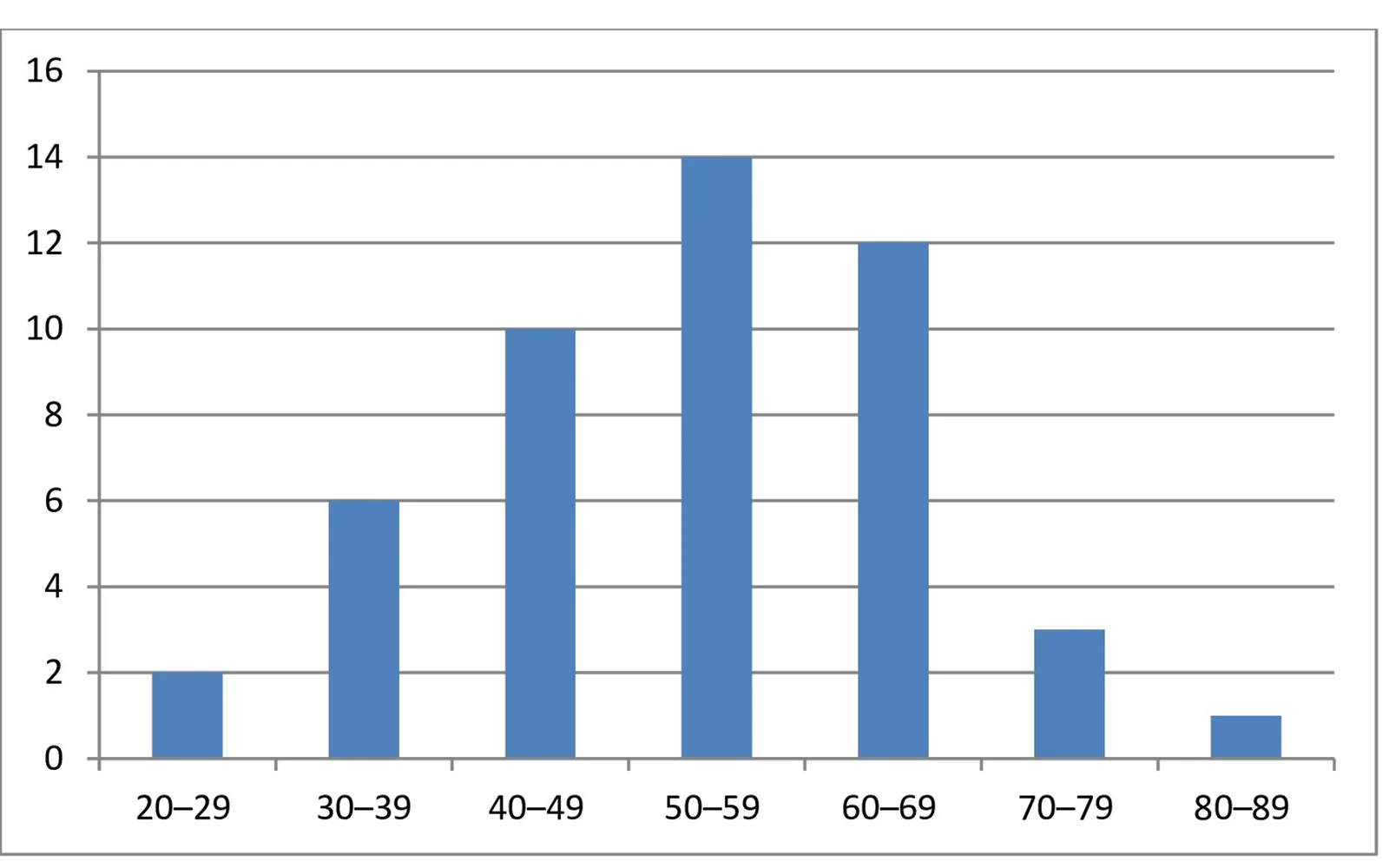
Overall age distribution of the thrombolysis group

The thrombolysis group (Group A) included 48 patients. Twenty-seven (56.3%) were female and 21 (43.7%) were male. The mean age was 53.25 years (SD 13.63), with a median age of 53.50 years (IQR 43.50-64.25). Age ranged from 21 to 82 years (Figure 3).

The anticoagulation-only group (Group B) included 12 patients. Six patients (50.0%) were female and six (50.0%) were male. The mean age was 64.1 years. Patients managed with anticoagulation alone were older than those treated with thrombolysis (mean age 64.1 years vs 53.3 years).

Physiological response to systemic thrombolysis

**Figure 4:**
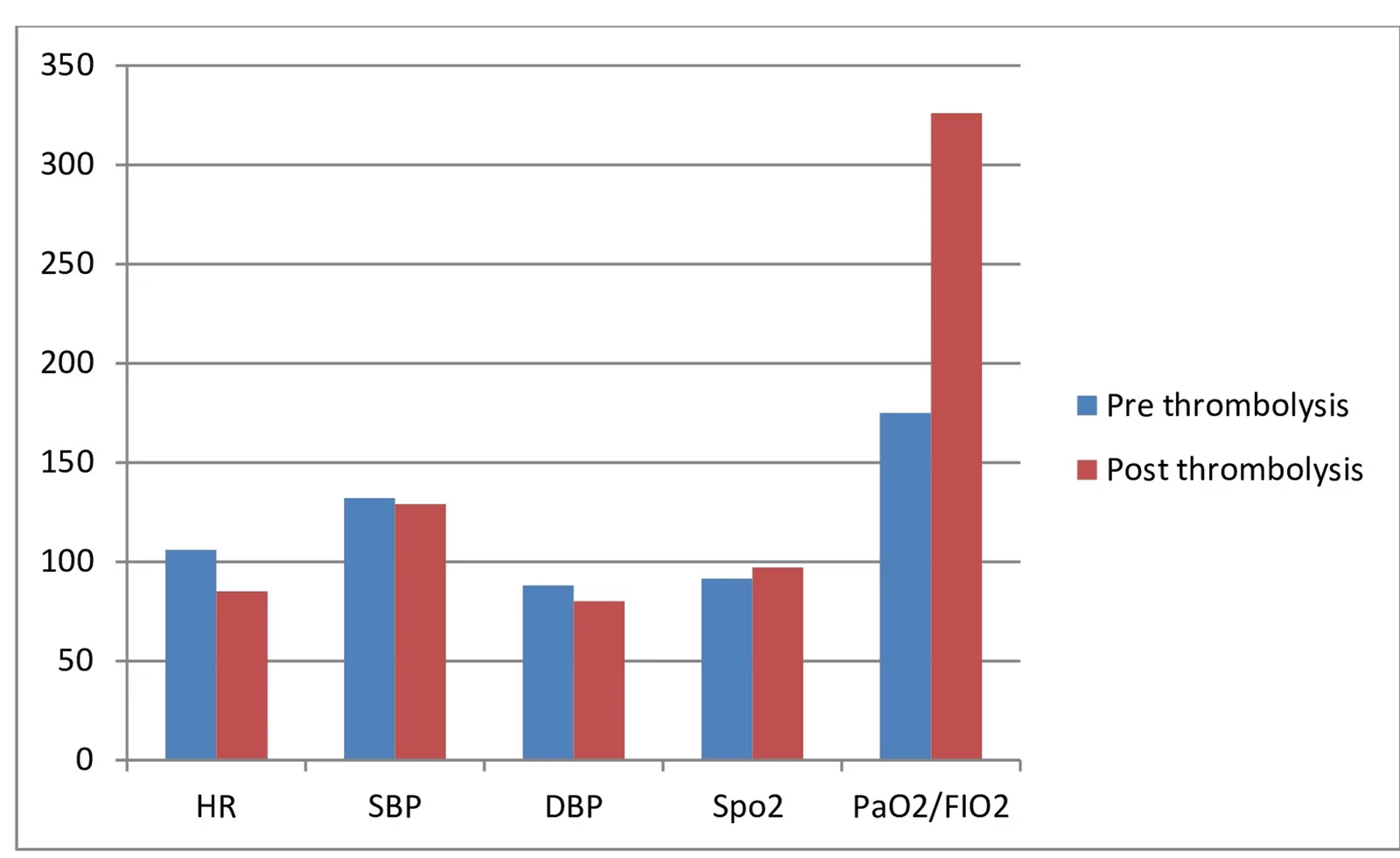
Average changes in the physiological parameters before and after thrombolysis

Among the 48 patients who received systemic thrombolysis, significant changes were observed in several physiological parameters following treatment (Figure 4). The mean heart rate decreased from 106 bpm before treatment to 85 bpm after treatment (p < 0.001). The mean oxygen saturation increased from 91.4% to 97.2% (p < 0.001). The mean PaO_2_/FiO_2_ ratio increased from 175 to 326 (p < 0.00001). Blood pressure remained stable following treatment, with mean systolic blood pressure changing from 132 mmHg to 129 mmHg and mean diastolic blood pressure from 88 mmHg to 80 mmHg (Table 2).

**Table 2:** Physiological response to systemic thrombolysis (group A, n = 48) Data are presented as arithmetic means ± SD. Statistical comparisons were performed using a two-tailed paired t-test. Significance threshold set at p < 0.05.

Clinical criterion	Baseline pre-treatment (mean ± SD)	Post-treatment (mean ± SD)	Test statistic (t-value)	Clinical significance / p-value
Heart rate (HR)	106 ± 14.2 bpm	85 ± 9.6 bpm	t = 8.42	Resolution of sinus tachycardia (p < 0.001)
Systolic blood pressure	132 ± 18.5 mmHg	129 ± 12.1 mmHg	t = 1.04	Maintenance of haemodynamic stability (p = 0.303)
Diastolic blood pressure	88 ± 11.3 mmHg	80 ± 8.7 mmHg	t = 2.11	Physiological normalization (p = 0.040)
Oxygen saturation (SpO_2_)	91.4% ± 3.1%	97.2% ± 1.4%	t = 11.15	Resolution of systemic hypoxia (p < 0.001)
PaO_2_/FiO_2_ ratio	175 ± 34.8	326 ± 45.2	t = 19.63	Reversal of acute respiratory compromise (p < 0.00001)

Echocardiographic evaluation of the right ventricular function

TAPSE Metrics

**Figure 5:**
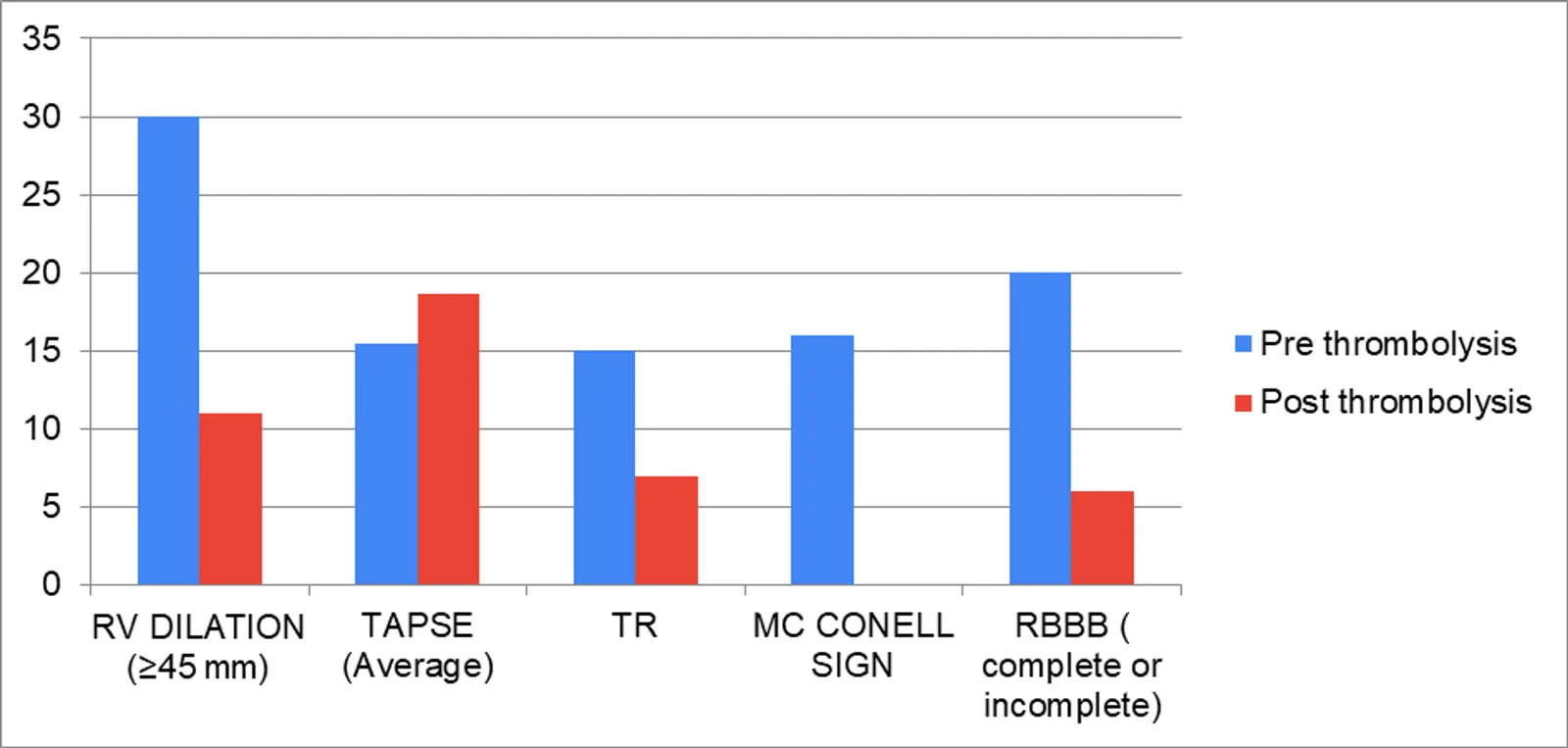
Average changes in the echocardiography parameters before and after thrombolysis

In patients with paired transthoracic echocardiograms (n = 6), the mean TAPSE significantly increased from 15.33 ±3.05 mm at baseline to 18.50 ±2.43 mm following systemic thrombolysis (t = 3.87, p = 0.0104) (Figure 5).

RV Chamber Geometry

Out of 18 patients with complete serial structural imaging, 17 (94.4%) exhibited severe RV dilation at baseline. Post-thrombolysis follow-up imaging confirmed the complete normalization of RV structural dimensions in seven of these patients (38.9%), establishing a significant structural reversal of chamber dilatation (McNemar’s χ2 = 5.85, p = 0.0156).

McConnell's Sign

Pathognomonic RV free wall hypokinesia with sparing of the apex (McConnell’s sign) was documented in 16 patients at presentation. Follow-up echocardiographic evaluation confirmed the 100% resolution of this sign across all evaluated patients post-thrombolysis (McNemar’s χ2= 14.06, p < 0.001). 

Treatment utilization trends and chronology

**Figure 6:**
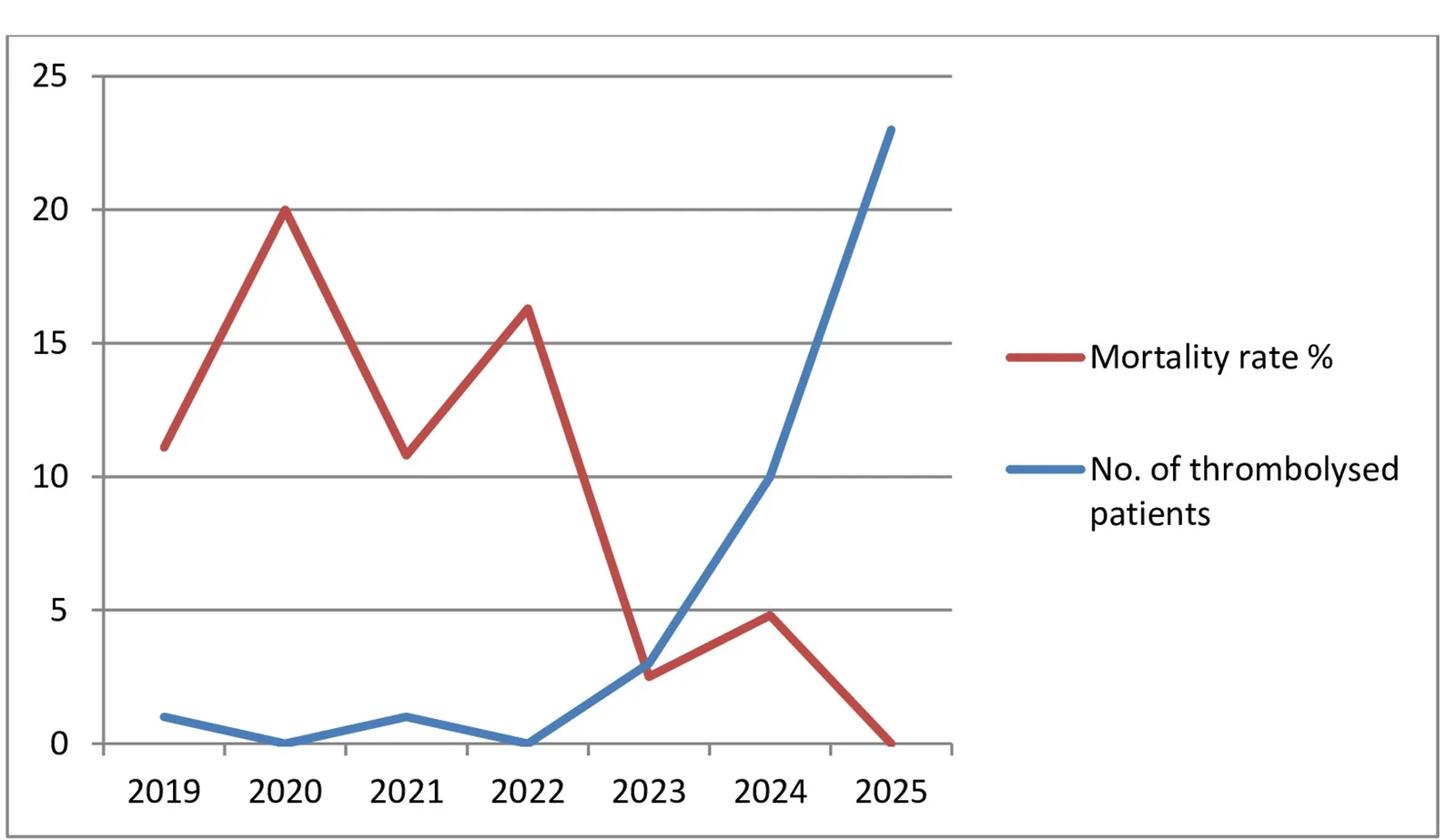
Correlation between the number of thrombolyzed patients/year and mortality rate

The clinical adoption of early systemic thrombolytic intervention expanded considerably over time (Figure 6). Annual deployment within intermediate-risk PE patients evolved as follows: one patient (2.1%) in 2019, zero (0.0%) in 2020, one (2.1%) in 2021, zero (0.0%) in 2022, three (6.3%) in 2023, 10 (20.8%) in 2024, 23 (47.9%) in 2025, and 10 (20.8%) during the first five months of 2026. Weight-adjusted bolus tenecteplase was administered to 32 (66.7%) patients, while 16 (33.3%) received conventional alteplase infusions. Upfront reperfusion was instituted within 24 hours of hospital presentation in 32 (66.7%) cases.

Characteristics of patients managed without thrombolysis

Group B included 12 patients managed with anticoagulation alone. Six patients (50.0%) were female and six (50.0%) were male. Mean age was 64.1 years. Reasons for withholding thrombolysis were categorized as absolute contraindications, relative contraindications, or clinician treatment decisions based on older treatment protocols.

Absolute Contraindications (n = 2; 16.7%)

One patient presented with an acute brainstem ischemic stroke (subsequently suffering a fatal cardiac arrest). A second patient presented with an active hematological malignancy complicated by concurrent severe trauma (survived).

Relative Contraindications (n = 3; 25.0%)

They comprised recent major surgery, severe polytrauma (n = 1; deceased via sudden arrest), active alcoholic hepatitis (n = 1; survived), and advanced age with unilateral right main artery thrombus (one patient, survived). In seven patients(n = 7; 58.3%), management reflected an earlier hospital treatment protocol in which thrombolysis was reserved for clinical deterioration; thrombolysis was withheld because they remained haemodynamically stable with mild hypoxaemia, although two patients suffered sudden, unpredicted fatal cardiac arrests despite initial respiratory improvement.

Clinical outcomes and safety

Clinical survival profiles differed significantly between the two cohorts. In Group A, 47 of 48 patients survived to discharge, representing an all-cause survival rate of 97.9% and a crude mortality rate of 2.1%. In Group B, eight of 12 patients survived, resulting in a survival rate of 66.7% and an all-cause mortality rate of 33.3%. Fisher's exact test confirmed that systemic thrombolysis was associated with a significant survival benefit over anticoagulation monotherapy (p = 0.0045).

The single mortality in Group A occurred in an 82-year-old female patient who developed an intracranial haemorrhage post-thrombolysis and subsequently developed secondary hospital-acquired pneumonia. This event represented the sole major haemorrhagic complication recorded across the entire intermediate-risk cohort (1/48; 2.1%). Minor bleeding in Group A included transient hematuria (n = 11; 22.9%) and self-limiting gingival bleeding (n = 3; 6.3%), all resolved spontaneously within 24 hours. No major bleeding events occurred in Group B (Table 3).

**Table 3:** Comparative outcomes and safety profiles of submassive pulmonary embolism (PE) management groups Data are presented as absolute numbers paired with percentages (N/%). Statistical analysis for survival and mortality was performed via Fisher's exact test; paired structural metrics were evaluated via t-tests and McNemar's tests. Significance threshold set at p < 0.05.

Outcome parameter	Group A: systemic thrombolysis (n = 48)	Group B: anticoagulation only (n = 12)	Statistical test statistic	Significance (p-value)
All-cause survival rate	97.92% (47/48)	66.67% (8/12)	Fisher's exact test value = 0.018	p = 0.0045 (highly significant)
All-cause mortality rate	2.08% (1/48)	33.33% (4/12)	Fisher's exact test value = 0.018	p = 0.0045
Direct cause (RV failure) mortality	0% (0/48)	33.33% (4/12)	Fisher's exact test value = 0.001	p = 0.0012
Major hemorrhage (ICH), n	1 (2.08%)	0 (0.00%)	Fisher's exact test value = 1.000	p = 1.0000
ICU-specific diatheses	Hematuria (n = 11) / gum bleed (n = 3)	0 / 0	Descriptive count	—
Functional RV recovery (TAPSE)	Significant improvement (+3.17 mm)	No data	Paired t = 3.87 (df = 5)	p = 0.0104 (pre- vs. post-thrombolysis data)
Structural RV resizing (dilation)	Significant reversal to normal	No data	McNemar's χ2 = 5.85 (df = 1)	p = 0.0156 (pre- vs. post-thrombolysis data)
McConnell's sign resolution	Complete reversal (16 to 0 cases)	No data	McNemar's χ2 = 14.06 (df = 1)	p < 0.001 (pre- vs. post-thrombolysis data)

Illustrative case series for some patients selected from the study cohort 

Case 1: Conservative Anticoagulation Management and Sudden Hemodynamic Collapse

**Figure 7:**
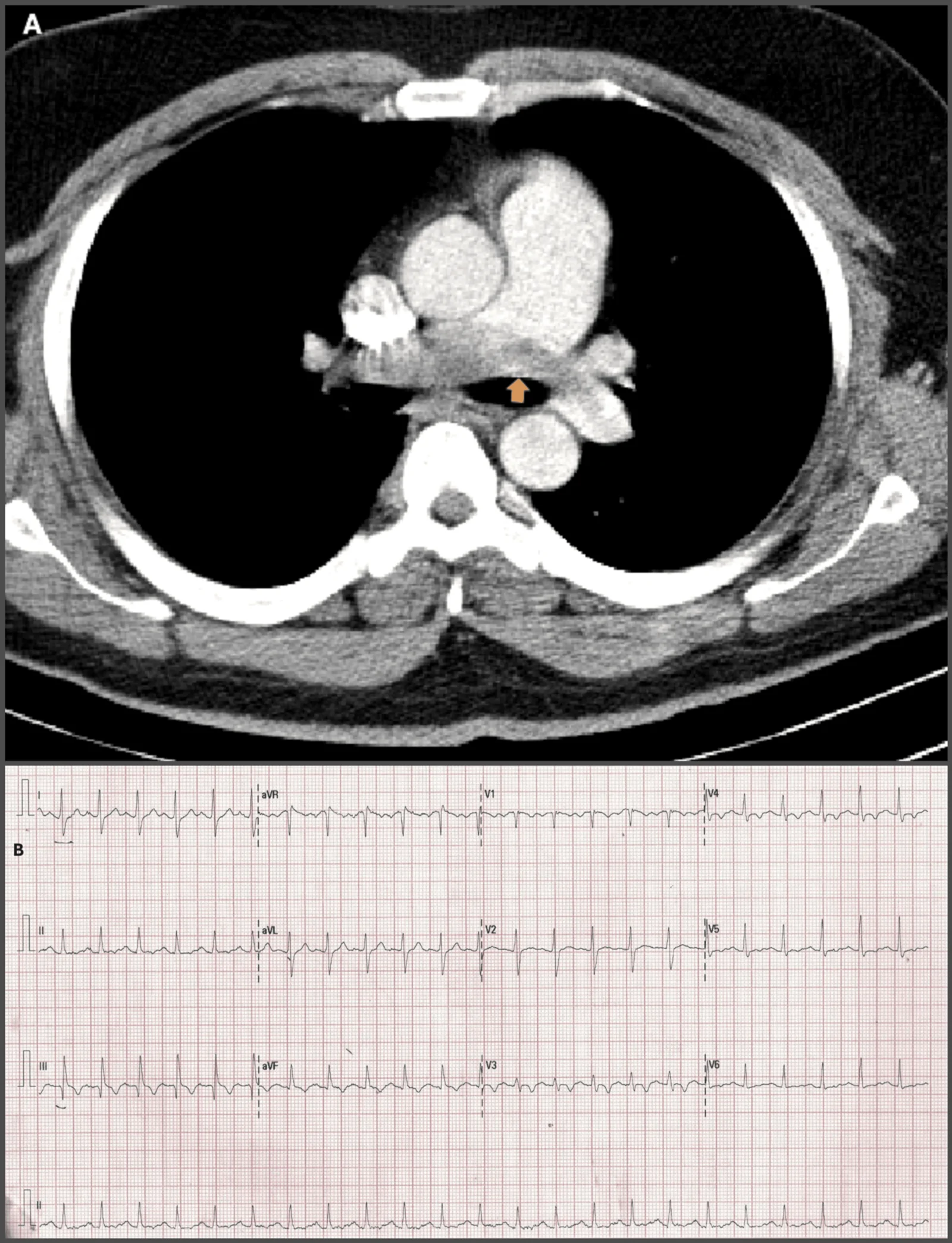
Case 1: A. Axial chest CT scan with contrast shows saddle pulmonary embolism (orange arrow). B. ECG shows sinus tachycardia, S1Q3T3, and inverted T waves in different leads.

A 36-year-old male presented with a submassive (intermediate-risk) PE a few weeks following a motor vehicle accident (Figure 7, Table 4). The accident had caused severe polytrauma requiring major orthopedic surgery for fracture fixation. Due to the high baseline risk of hemorrhage and the patient’s initial stable clinical appearance, systemic thrombolysis was deferred, and he was managed with therapeutic anticoagulation. However, despite continuous monitoring and normal physiological parameters, the patient suffered a sudden, unpredicted fatal cardiac arrest without any prior signs of clinical deterioration.

**Table 4:** Vital signs and monitoring matrix (Case 1)

Parameter observed	Baseline values	Standard reference range	Clinical significance	
Respirations	15-17 breaths/min	12-20 breaths/min	Normal resting respiratory rate	
Oxygen saturation (SpO₂)	≥96% (on supplemental O_2_)	95-100%	Maintained via oxygen therapy	
Blood pressure	100/72 mmHg to 129/88 mmHg	90/60 to 120/80 mmHg	Normotensive to pre-hypertensive	
Pulse (heart rate)	78-129 bpm	60-100 bpm	Intermittent sinus tachycardia	

Case 2: frontline systemic thrombolysis and rapid physiological reversal

**Figure 8:**
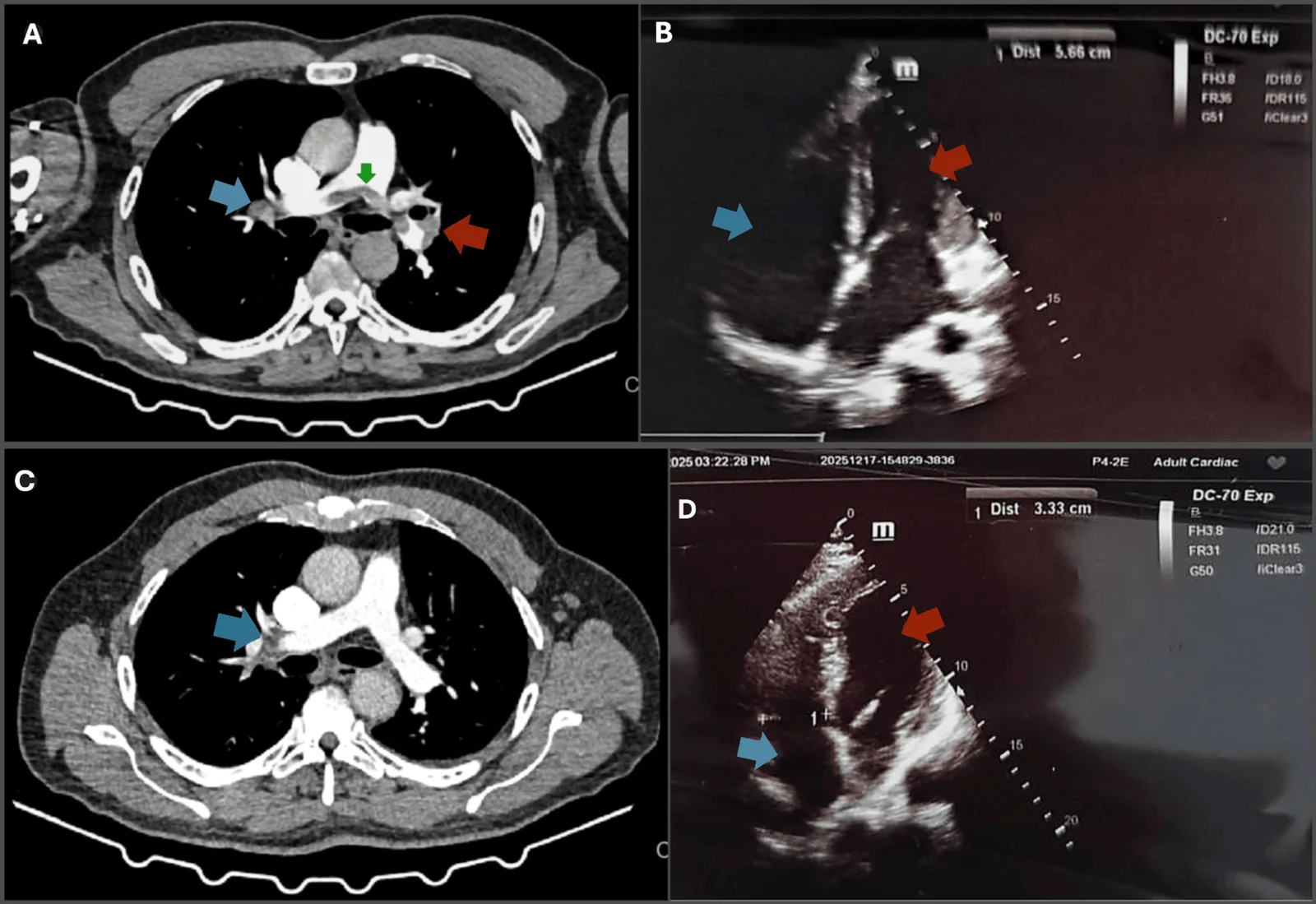
Case 2: A. Pre-thrombolysis CT scan with IV contrast axial plane shows saddle pulmonary embolism (green arrow), right (blue arrow), and left (orange arrow) pulmonary artery emboli. B. Pre-thrombolysis bedside echocardiography, apical four-chamber view showed dilated RV (blue arrow) dimensions. > left ventricle (LV) (orange arrow), basal RV: 56 mm. C. Post-thrombolysis CT scan with IV contrast shows complete resolution of the saddle and left pulmonary artery emboli with partial resolution of the right pulmonary artery embolus (blue arrow). D. Post-thrombolysis bedside echocardiography; apical four-chamber view showed normal RV (blue arrow). < LV dimension (orange arrow), RV basal dimension 33 mm.

A 63-year-old male presented with an acute intermediate-risk PE and received immediate systemic thrombolysis upon admission (Figure 8). Pre-treatment evaluations showed a massive clot burden and severe right ventricular strain, which rapidly reversed following pharmacological reperfusion.

Case 3: prompt reperfusion in an elderly patient

**Figure 9:**
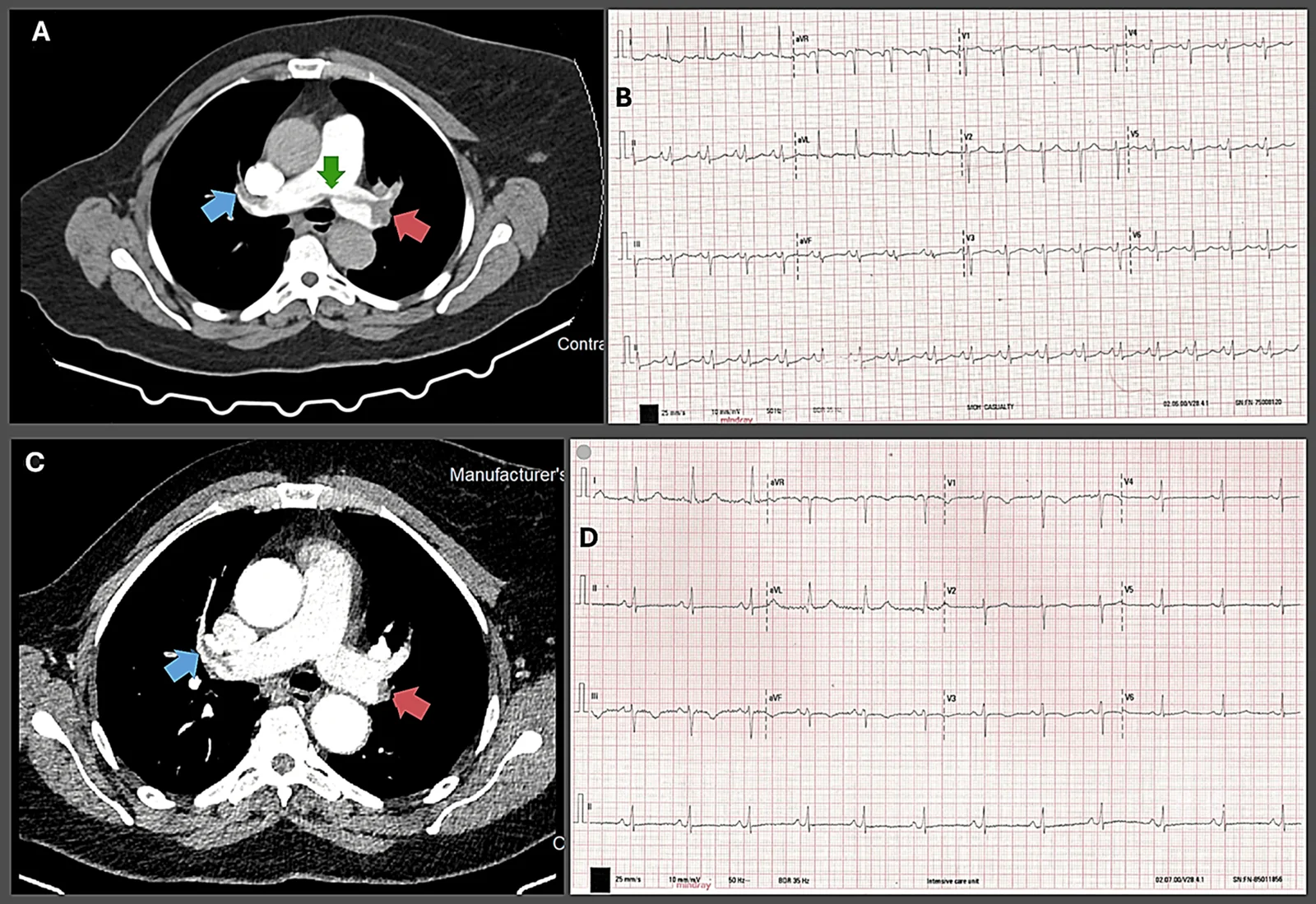
Case 3: A. Pre-thrombolysis axial CT scan with contrast shows saddle pulmonary embolus detected at the bifurcation of the pulmonary artery (green arrow), extending to the right main pulmonary artery (blue arrow) and left pulmonary artery (orange arrow). B. Pre-thrombolysis ECG shows sinus tachycardia. C. Post-thrombolysis CT with contrast axial plane shows complete resolution of the saddle embolus with partially resorbed right main pulmonary artery (blue arrow) and left pulmonary artery (orange arrow). D. Post-thrombolysis ECG shows resolution of the tachycardia.

A 73-year-old female presented with an acute intermediate-risk PE and was treated with systemic thrombolysis upon hospital admission (Figure 9).

Case 4: delayed reperfusion following medical stabilization

**Figure 10:**
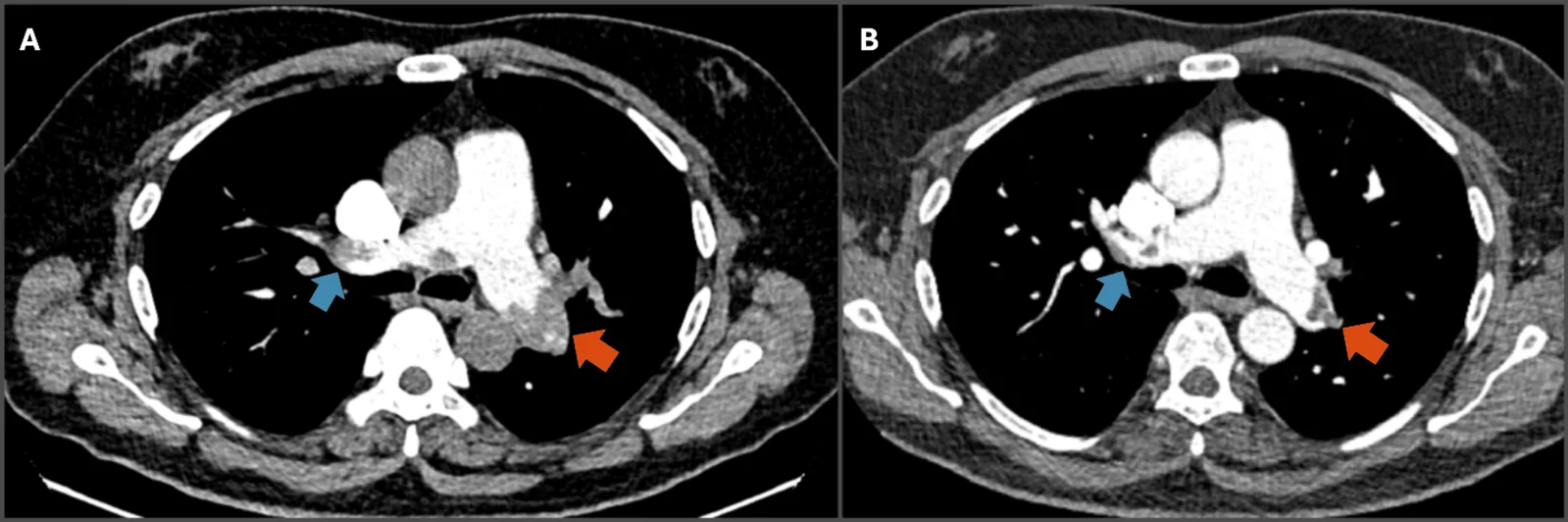
Case 4: A. Pre-thrombolysis axial CT scan with contrast shows bilateral pulmonary embolism occluding the right main pulmonary artery (blue arrow) and the left main pulmonary artery (orange arrows). B. Post-thrombolysis axial CT scan shows significant recanalization of the right main pulmonary artery (blue arrow) and the left pulmonary artery (orange arrow).

A 52-year-old male with a recent history of bilateral ureteral lithiasis underwent lithotripsy and double-J catheter insertion. He was admitted with a recurrent urinary tract infection complicated by severe anemia and acute kidney injury. Following stabilization with intravenous fluids, antibiotics, and blood transfusions, he had persistent tachycardia, tachypnea, and hypoxia. A CT angiogram performed four days after admission confirmed a submassive PE (Figure 10). He received systemic thrombolytic therapy, which led to a rapid and complete resolution of his clinical symptoms and vital signs.

Case 5: corrective delayed thrombolysis in severe anemia

**Figure 11:**
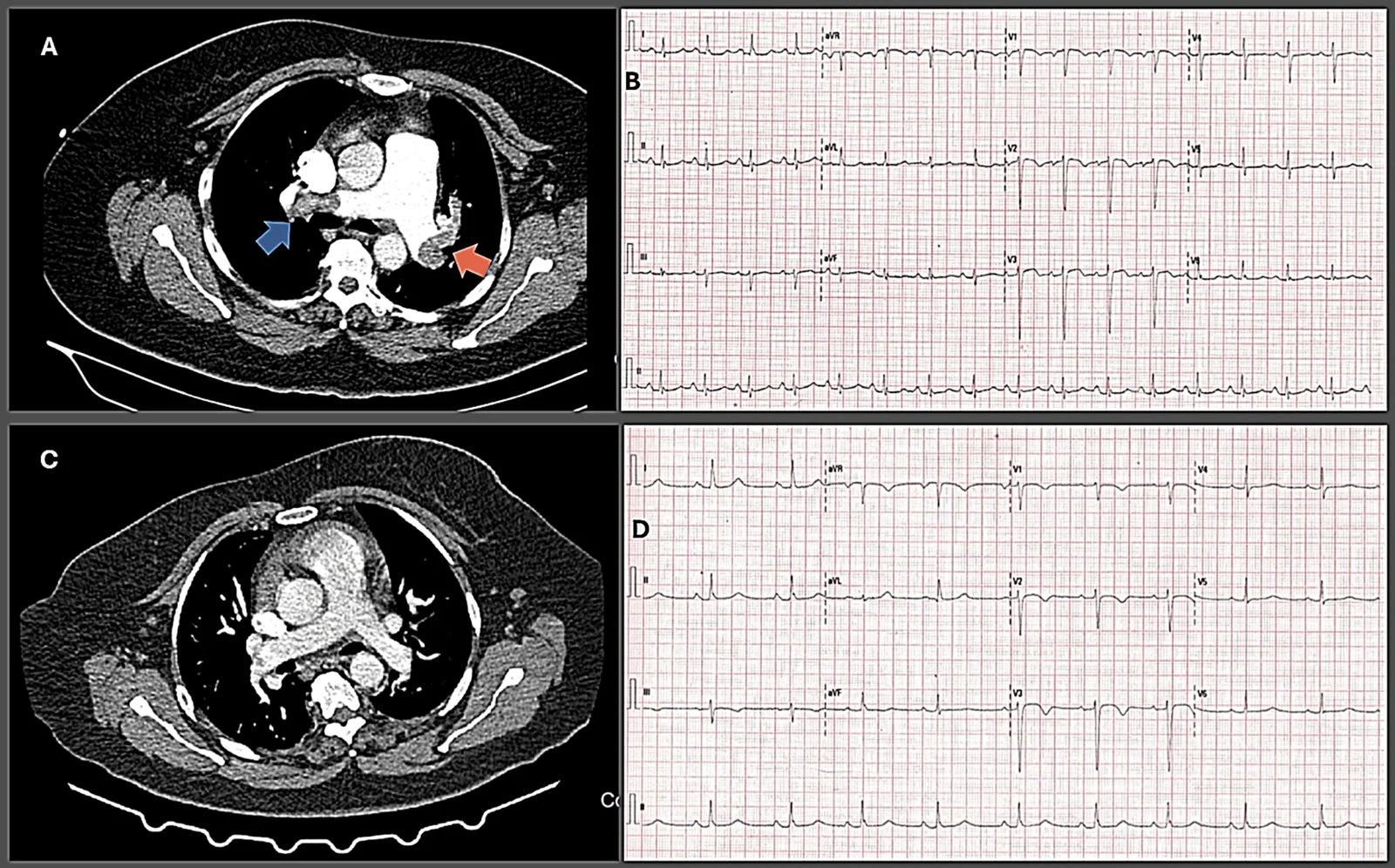
Case 5: A. Pre-thrombolysis axial CT angiogram shows bilateral pulmonary embolism, involving the right main pulmonary artery (blue arrow) and left main pulmonary artery (orange arrow). B. Pre-thrombolysis ECG shows borderline tachycardia. C. Post-thrombolysis axial CT angiogram shows resolved pulmonary embolism bilaterally. D. Post-thrombolysis ECG shows resolution of tachycardia, with persistent inverted T wave in anterior leads.

A 53-year-old female with a history of acute PE in 2021 (post-COVID-19, managed with anticoagulation alone) was readmitted in 2025 with severe hypoxia and profound anemia (hemoglobin: 5.0 g/dL) whereby she was diagnosed with recurrent intermediate-risk PE. Thrombolysis was initially deferred to investigate and correct the anemia, which was subsequently diagnosed as a heavy Strongyloides stercoralis infestation. She was placed on anticoagulation monotherapy, received blood transfusions, and was treated with albendazole. Despite these measures, she remained severely hypoxic, with her oxygen saturation (SpO_2_​) dropping to 82% whenever supplemental oxygen was removed. Due to persistent respiratory failure, systemic thrombolysis was administered one week after admission. This delayed intervention led to a complete resolution of her symptoms and normalization of her vital signs (Figure 11).

Case 6: radiological-clinical dissociation in a young female patient

**Figure 12:**
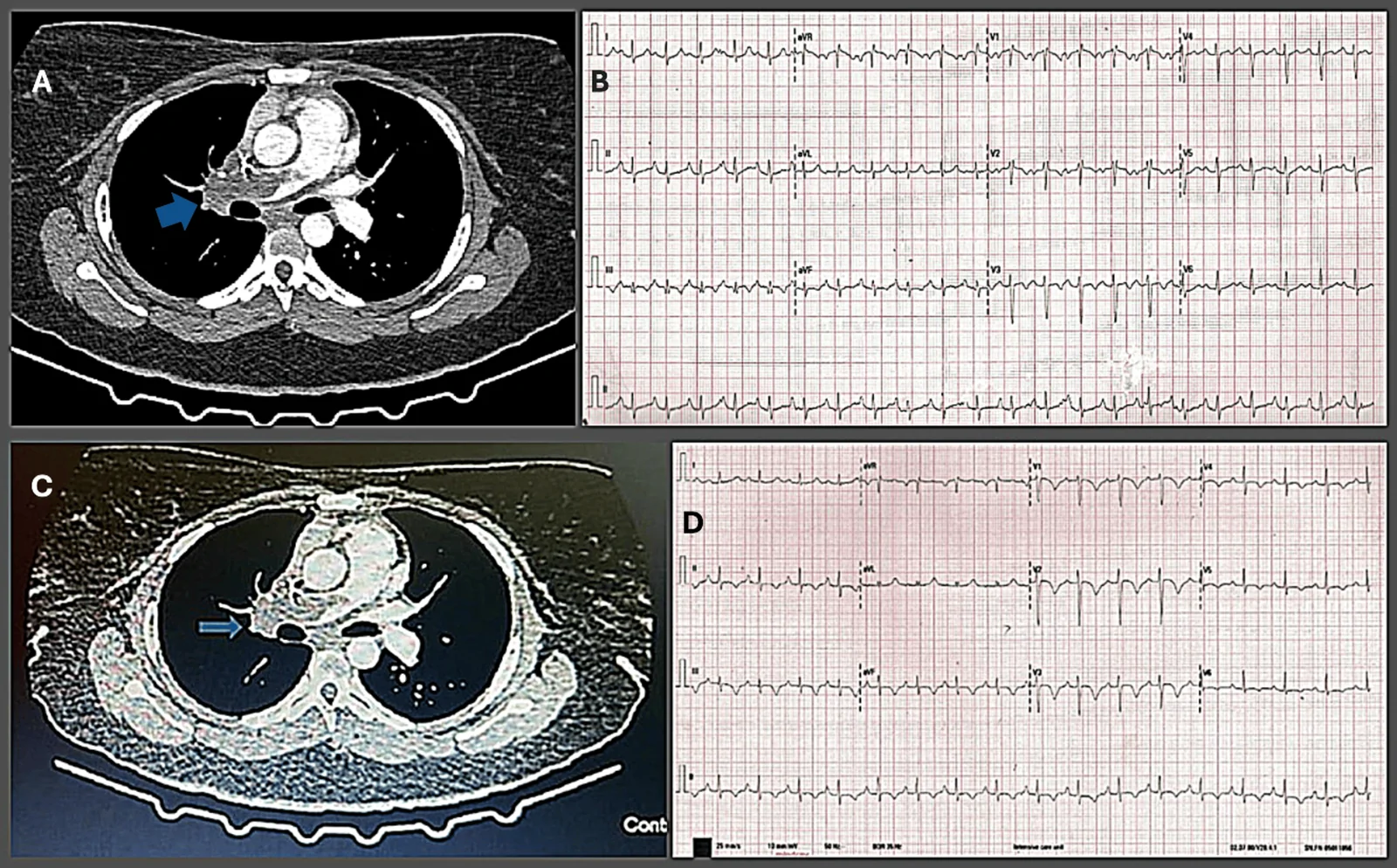
Case 6: A. Pre-thrombolysis chest CT angiogram shows the right main pulmonary artery thrombus (blue arrows). B. Pre-thrombolysis shows sinus tachycardia and incomplete right bundle branch block (RBBB). C. Post-thrombolysis chest CT angiogram shows minimal resolution of the right main pulmonary artery thrombus (blue arrow). D. Improved tachycardia with resolution of RBBB and persistent widespread T-wave abnormality.

A 21-year-old female was admitted with an acute intermediate-risk PE and treated with frontline systemic thrombolysis. A follow-up CT angiogram showed minimal change in the physical thrombus (Figure 12). However, she achieved rapid physiological recovery, with immediate normalization of her heart rate and respiratory rate (Table 5), along with complete resolution of right ventricular strain on both ECG and echocardiography (Figure 12). Table 6 presents in detail the evolution of ABG findings and PAO_2_/FiO_2_ ratios following thrombolysis in selected illustrative cases from the study cohort.

**Table 5:** Vital signs and monitoring matrix (Case 6)

Monitoring parameter	Pre-thrombolysis	Post-thrombolysis	Standard reference range	Clinical outcome	
Heart rate	124 bpm	82 bpm	60–100 bpm	Resolution of tachycardia	
Respiratory rate	26 breaths/min	16 breaths/min	12–20 breaths/min	Resolution of tachypnea	
Oxygen saturation (SpO₂)	89% (room air)	98% (room air)	95–100%	Reversal of systemic hypoxia	
Systolic blood pressure	118 mmHg	122 mmHg	90–120 mmHg	Stable haemodynamics	
Diastolic blood pressure	76 mmHg	80 mmHg	60–80 mmHg	Stable haemodynamics	

**Table 6:** Evolution of arterial blood gas (ABG) findings and PAO2/FiO2 ratios following thrombolysis in selected illustrative cases from the study cohort.

Case 2	Pre-thrombolysis levels	Post-thrombolysis levels	Standard reference range
PH	7.45	7.5	7.35-7.45
PCO2	33.3	33.4	35.0-45.0 mmHg
PaO2	142	88.7	75.0-100.0 mmHg
HCO3	24.1	25.8	22.0-26.0 mmol/L
FiO2	0.9	0.21	
PaO2/FiO2 ratio	157	422	>400
Case 4	Pre-thrombolysis levels	Post-thrombolysis levels	Standard reference range
PH	7.49	7.5	7.35-7.45
PCO2	32.2	36.3	35.0-45.0 mmHg
PaO2	56.1	65	75.0-100.0 mmHg
HCO3	24.6	28	22.0-26.0 mmol/L
FiO2	0.6	0.21	
PaO2/FiO2 ratio	93	309	>400
Case 5	Pre-thrombolysis levels	Post-thrombolysis levels	Standard reference range
PH	7.49	7.46	7.35-7.45
PCO2	31.6	33.6	35.0-45.0 mmHg
PaO2	86.7	73.2	75.0-100.0 mmHg
HCO3	23.9	23.8	22.0-26.0 mmol/L
FiO2	0.99	0.21	
PaO2/FiO2 ratio	88	349	>400
Case 6	Pre-thrombolysis levels	Post-thrombolysis levels	Standard reference range
PH	7.5	7.43	7.35-7.45
PCO2	39.7	24.2	35.0-45.0 mmHg
PaO2	64.8	89.6	75.0-100.0 mmHg
HCO3	30.8	15.6	22.0-26.0 mmol/L
FiO2	0.6	0.21	
PaO2/FiO2 ratio	108	427	>400

## Discussion

This study provides a national overview of acute PE in the Seychelles between 2019 and 2025 and reports outcomes among patients with intermediate-risk PE managed with systemic thrombolysis or anticoagulation alone.

The number of documented PE cases increased during the study period, reaching a peak in 2024. Several factors may have contributed to this trend, including increased awareness of venous thromboembolism, wider use of computed tomography pulmonary angiography (CTPA), and improvements in case identification following implementation of an electronic medical record system. Interpretation of temporal trends should also consider potential changes in documentation and reporting practices during the study period.

Annual mortality varied substantially across years. The higher mortality observed in 2020 should be interpreted cautiously because data collection during the COVID-19 pandemic was incomplete and healthcare delivery was affected by pandemic-related disruptions. In addition, COVID-19 has been associated with an increased risk of thromboembolic disease and may have influenced the characteristics of patients presenting during this period [13,14].

The subgroup analysis focused on patients with intermediate-risk PE, a population for whom optimal management remains uncertain. Current international guidelines recommend systemic thrombolysis for high-risk PE but do not recommend routine thrombolysis for all patients with intermediate-risk PE because potential benefits must be balanced against the risk of major bleeding and intracranial haemorrhage. Previous randomized trials and meta-analyses have demonstrated reductions in haemodynamic deterioration following thrombolytic therapy, although effects on overall mortality remain less consistent [6,15].

Because our sample size limits the use of advanced multivariable modeling to fully control for these confounding variables, our subgroup comparisons are presented strictly as exploratory, association-based observations rather than a direct, unconfounded evaluation of thrombolysis efficacy.

In the past, local protocols relied strictly on conservative anticoagulation monotherapy with close monitoring, escalating to rescue thrombolysis only upon overt secondary deterioration. However, this stringent delayed approach frequently encountered unpredictable cardiac arrests that occurred without perceptible warning signs, prompting a deliberate transition toward early systemic thrombolytic intervention.

In the present study, lower observed mortality was recorded among patients treated with systemic thrombolysis compared with anticoagulation alone. Patients receiving thrombolysis also demonstrated improvements in physiological parameters, including heart rate, oxygen saturation, and PaO_2_/FiO_2_ ratio, together with favourable changes in echocardiographic markers of right ventricular function [15]. These findings suggest that thrombolysis may be associated with early haemodynamic and right ventricular recovery in selected patients with intermediate-risk PE.

Tenecteplase became the most frequently used thrombolytic agent after its inclusion in the Seychelles National Essential Medication List in mid 2024. Its single-bolus administration simplified treatment delivery compared with prolonged infusion protocols and was readily incorporated into local clinical practice. Because this study was not designed to compare tenecteplase with alteplase directly, no conclusions regarding comparative efficacy or safety can be drawn.

Bleeding remains an important consideration when selecting patients for thrombolytic therapy. One patient experienced intracranial haemorrhage following thrombolysis, whereas minor bleeding events were generally self-limited and managed conservatively. These findings are consistent with the established balance between potential benefits and bleeding risks associated with systemic thrombolysis.

An additional observation from this study relates to the timing of thrombolytic therapy. Although most patients received treatment within 24 hours of presentation, selected patients underwent delayed thrombolysis after stabilization of temporary contraindications or correction of reversible clinical factors. Favourable outcomes were observed in several of these cases. However, the present study was not designed to determine an optimal treatment window, and no firm conclusions regarding delayed thrombolysis can be drawn [16].

Some patients demonstrated substantial clinical and echocardiographic improvement despite limited radiological change on follow-up imaging.

This observation suggests that clinical recovery may not always parallel radiological findings and highlights the importance of integrating physiological, imaging, and clinical assessments when evaluating treatment response.

This study has some limitations that should be considered when interpreting its findings. First, its retrospective, observational design makes it susceptible to historical charting bias and incomplete records. These challenges were particularly evident during the 2020 COVID-19 pandemic wave and the national EMR transition in late 2024. Second, the sample sizes - especially within the anticoagulation monotherapy group (n = 12) - are small, which limits the statistical power needed to detect less common safety outcomes or perform multivariable modeling.

Third, supply chain constraints common to remote island nations resulted in frequent stockouts of key cardiac biomarkers, such as cardiac troponins and pro-BNP, which prevented us from incorporating them into our risk assessment. Finally, because treatment decisions were left to the judgement of the attending clinicians, there is a risk of selection bias; older or more fragile patients may have been preferentially assigned to the conservative anticoagulation group. 

## Conclusions

This study provides the first comprehensive national assessment of acute PE epidemiology, clinical trends, and management outcomes in the Republic of Seychelles. Over a seven-year period, acute PE presented a significant clinical burden nationwide, with an increase in identified cases that highlights the impact of improved diagnostic access and the implementation of electronic medical records.

In resource-limited public healthcare settings that lack advanced interventional capabilities like catheter-directed mechanical thrombectomy or surgical embolectomy, systemic thrombolytic therapy represents a highly effective frontline strategy for carefully selected patients with intermediate-risk PE. 

Furthermore, weight-adjusted single-bolus tenecteplase provides an operationally efficient and effective therapeutic option that is well-suited for small-island healthcare infrastructure. Our clinical experience also suggests that a broader therapeutic window of up to seven days can be considered for delayed thrombolysis, provided that temporary contraindications have been stabilized and the patient's clinical condition warrants intervention, although this is an observation that needs more controlled clinical trials to determine the optimum time window for thrombolysis.

Ultimately, these findings highlight the clinical value of an early, systematic thrombolysis protocol to prevent unpredictable cardiac collapse and optimize outcomes for intermediate-risk PE in settings where advanced endovascular resources are unavailable.
